# Hybrid Molecular Dynamics for Elucidating Cooperativity Between Halogen Bond and Water Molecules During the Interaction of p53-Y220C and the PhiKan5196 Complex

**DOI:** 10.3389/fchem.2020.00344

**Published:** 2020-05-07

**Authors:** Tian-ge Dong, Hui Peng, Xue-feng He, Xiaocong Wang, Jun Gao

**Affiliations:** Hubei Key Laboratory of Agricultural Bioinformatics, College of Informatics, Huazhong Agricultural University, Wuhan, China

**Keywords:** quantum mechanical/molecular mechanical molecular dynamics, cooperativity, halogen bond, hydrogen bond, p53 protein

## Abstract

The cooperativity between hydrogen and halogen bonds plays an important role in rational drug design. However, mimicking the dynamic cooperation between these bonds is a challenging issue, which has impeded the development of the halogen bond force field. In this study, the Y220C–PhiKan5196 complex of p53 protein was adopted as a model, and the functions of three water molecules that formed hydrogen bonds with halogen atoms were analyzed by the simulation method governed by the hybrid quantum mechanical/molecular mechanical molecular dynamics. A comparison with the water-free model revealed that the strength of the halogen bond in the complex was consistently stronger. This confirmed that the water molecules formed weak hydrogen bonds with the halogen atom and cooperated with the halogen atom to enhance the halogen bond. Further, it was discovered that the roles of the three water molecules were not the same. Therefore, the results obtained herein can facilitate a rational drug design. Further, this work emphasizes on the fact that, in addition to protein pockets and ligands, the role of voids should also be considered with regard to the water molecules surrounding them.

## Introduction

Halogen bonds have become a trending subject for studies aiming at contributing to rational drug design (Lu et al., 2012). These bonds are an important non-covalent interaction in a protein–ligand binding process (Lu et al., 2009) and are similar to hydrogen bonds (Metrangolo et al., 2005). Although the contributions of different parameters vary, the electrostatic property, dispersion, polarization, and charge transfer effects are all known to contribute to the halogen bond interaction (Hanna, 1968; Riley et al., 2013; Esrafili et al., 2017). Previously, a σ-hole model was developed to characterize halogen bonds, wherein a crown of positive charge on the outer side of a halogen atom could form a halogen bond and a belt of negative charge surrounding the halogen atom could form a hydrogen bond (Clark, 2013, 2017; Politzer et al., 2013, 2017). Therefore, the co-existence of both halogen as well as hydrogen bonds due to the presence of a σ-hole in a halogen atom is one of the important characteristics of halogen bonds (Li et al., 2008; Alkorta et al., 2010; Zhao et al., 2011; Yan et al., 2014; Zhou et al., 2016; Molina et al., 2017; Ciancaleoni, 2018).

In recent years, numerous studies have reported the positive cooperativity between halogen bonds and hydrogen bonds (Li et al., 2008; Grabowski, 2013; Wu et al., 2013; Esrafili and Mousavian, 2017; Esrafili and Vakili, 2017; Carlsson et al., 2018) as well as the effects of the substituents on the cooperativity of halogen bonds (Solimannejad et al., 2013). In view of the potential application prospects of halogen bonds in the field of drug design, Adasme-Carreno et al. (2016) performed calculations on 126 complexes of drug-like molecules; consequently, a positive cooperativity effect was observed in N-methylacetamide complexes with di-, tri-, and tetrafluoro-iodobenzenes, which led to the strengthening of halogen bonds. The water molecules are ubiquitous in protein complexes; particularly, those in close proximity to halogen bonds exhibited reciprocal interactions (De Santis et al., 2018). For example, this was observed during the enhancement of anion recognition (Langton et al., 2014) and strengthening binding affinity for CK2 ligands containing halogen atoms (Deepa, 2017; Deepa et al., 2018).

Robinson et al. (2016) employed explicit solvent molecular dynamics and solvent thermodynamic analysis in solvent-exposed ligand modifications to improve ligand selectivity, which was determined by water thermodynamics. Breiten et al. (2013) reported that the water molecules fill the active site of the protein and surround the ligand and are as important as the ligands that possess biomolecular recognition and determine the thermodynamics of bindings. Therefore, we are interested in validating the existence of the hydrogen bonds that are formed by the water molecules with nearby halogen atoms, elucidating the cooperative effects between the hydrogen and the halogen bonds, and investigating the effects of the thermodynamic behavior of water molecules on the stability of the halogen bonds.

In order to be able to reproduce the σ-hole characteristics in molecular modeling, several force fields for halogen bonds have been developed, such as extra-point charge (Ibrahim, 2011), force field for biological halogen bonds (Carter et al., 2012), OPLS-AAx and OPLS/CM1Ax (Jorgensen and Schyman, 2012), and multipole- (El Hage et al., 2016) and quantum-based generalized empirical potential (Santos et al., 2017). The polarizable ellipsoidal force field (Du et al., 2013; Liu et al., 2015) represented the anisotropic charge distribution with the combination of a negatively charged sphere and a positively charged ellipsoid. This model could correctly reproduce the potential energy surface of halogen bonds at the MP2 level. However, the performances of these force fields have not been completely tested with respect to the cooperative systems of hydrogen and halogen bonds; this is partially because the dynamics governing the cooperativity of the hydrogen and the halogen bonds are still unknown (Evangelisti et al., 2011; Chen et al., 2018; Lu et al., 2019).

**Figure 1:**
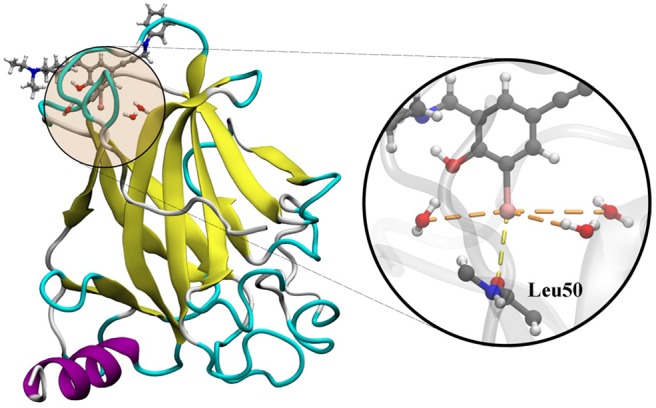
Crystal structure of the Y220C mutant of the p53 core domain bound to a stabilized small molecule PhiKan5196 (PDB ID: 4AGQ), in which the protein is shown as its secondary structure and the ligand is illustrated by the ball-and-stick model. The major interactions of P96 with Leu50 and three water molecules are indicated.

The utilization of the Y220C mutant of the p53 tumor suppressor with a ligand complex is a good approach to study the dynamics of the cooperativity of the hydrogen and the halogen bonds (Yang et al., 2019). In several cancers, p53 is inactivated by the direct mutation or perturbation of its associated pathways. The Y220C mutant destabilized p53 and generated a drugable surface crevice, which has led to approximately 75,000 new cancer cases per year. Wilcken et al. (2012) developed a strategy that utilized halogen bonds to stabilize the mutant with small molecules, which use halogen-enriched fragment libraries as the starting points. After a careful screening of their solved structures, we selected the Y220C–PhiKan5196 complex (PDB ID: 4AGQ) as the model. As shown in the crystal structure (Figure 1), a halogen bond was formed between the iodine atom of PhiKan5196 (P96) and the carbonyl oxygen of Leu50. Three water molecules were observed to be around the iodine atom, which has not been addressed previously. Therefore, it is necessary to explore the existence of hydrogen bonds that have been formed by these water molecules by incorporating iodine atoms and observing their cooperative effects with the existing halogen bonds.

In this work, we employed a method governed by hybrid quantum mechanical molecular mechanical molecular dynamics (QM/MM MD) to simulate the Y220C–PhiKan5196 complex; this method considered the polarization effects of the halogen and the hydrogen bonds and was successful in striking a balance between computational demand and accuracy (Ke et al., 2009; Ahmadi et al., 2018; Awoonor-Williams and Rowley, 2018; Steinmann et al., 2018; Visscher et al., 2018). For comparison purposes, we designed two models, wherein one comprised three water molecules (model 2) and the other was rendered devoid of any water molecule by the removal of three water molecules in the QM region (model 1) (see “MATERIALS AND METHODS”). We compared the dynamic performance of halogen bonds between the two models and concluded that the hydrogen bonds formed by the three spatial correlated water molecules and the iodine atom were able to not only synergize but also strengthen the halogen bond. Furthermore, the contribution of each hydrogen bond to the halogen bond was observed to be different.

## Materials and Methods

### Computational Methods and Model Setup

**Figure 2:**
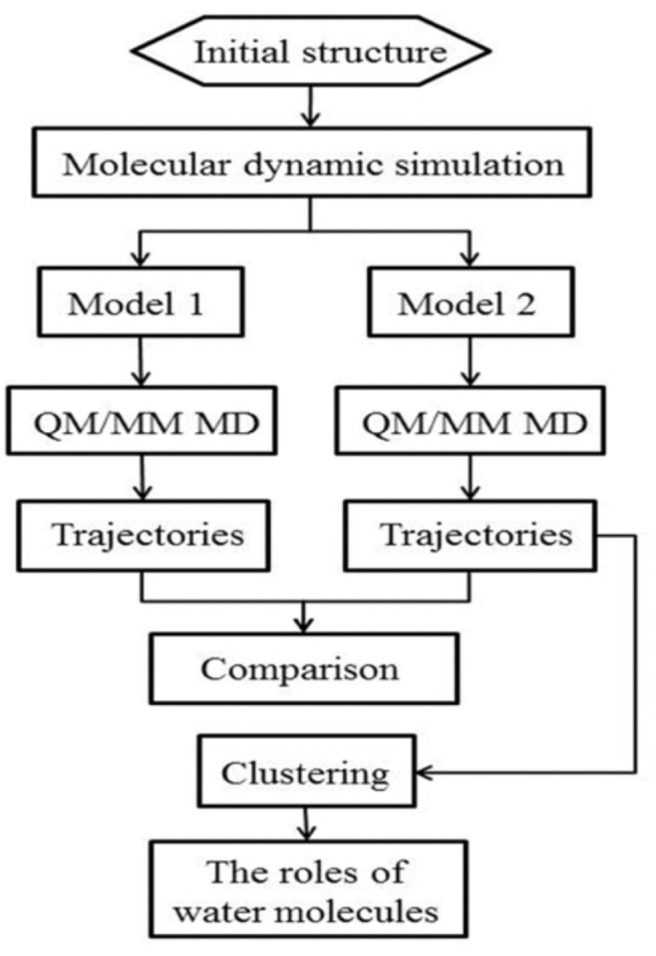
Schematic illustration of the workflow of the system setup and the QM/MM MD simulation.

The workflow of this study is shown in Figure 2. Starting from the crystal structure, we first carried out the classical molecular dynamic simulation to render the system equalized; two models were built for the QM/MM MD simulation. The trajectories were then classified for further analysis. The specific details of the method are described in the following discussion.

The preliminary structure of the classical MD simulation was selected from the crystal structure (PDB ID: 4AGQ); this structure was of the p53 core domain mutant, Y220C, which was bound to the stabilizing small molecule of PhiKan5196 (P96). It comprised two biological units: chain A and chain B; chain A was selected and chain B was manually deleted. The AMBER ff14SB force field (A. Maier et al., 2015) was used and the parameters of the ligands were derived from the generalized AMBER force field (Wang et al., 2004). The unbonded model (Pang, 1999) was applied to zinc and the surrounding amino acids. The complex was solvated in a cubical TIP3P water box with periodic boundary conditions, with a margin of at least 10Å from any edge of the box to any atom of the complex. The solvated systems were neutralized by adding Na^+^ and Cl^−^ ions and the final concentration for the solvated system was observed to be 0.15 M.

The system energy was minimized through conjugate gradient minimization. After that, the system was heated to 300 K at a constant volume over 2 ns. A 5-ns MD simulation was extended as production. An integral time step of 2 fs was used, and electrostatics were treated by the particle-mesh Ewald method (Essmann et al., 1995) with a 12-Å Coulomb interaction distance cutoff; further, the cutoff for the van der Waals interactions was set to 12 Å. The simulation was performed using the NAMD package (Phillips et al., 2005).

### QM/MM MD Simulation

**Figure 3:**
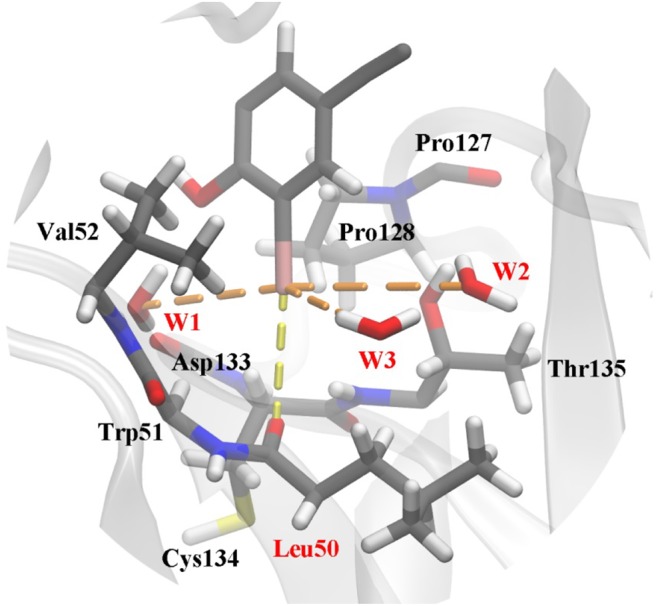
Structure of the active site in p53 Y220C that is used as the QM region in QM/MM MD simulations. The protein is shown as a gray cartoon representation and selected residues are highlighted as stick models. The halogen bond between the iodine and the carbonyl oxygen of Leu50 is indicated by a yellow dash line. Additional polar interactions between iodine and water are shown as orange dash lines. Water 1 to water 3 were named as W1 to W3 in this work.

The frame of the production trajectory that had the smallest root mean square deviation value with respect to the crystal structure was selected for the QM/MM MD simulation. We set up two models in this work. For model 2, the QM region was composed of the following atoms: all the atoms of Cys134, all side chain atoms and some of the backbone atoms of Val52, Leu50, Thr135, and Pro128, all the backbone atoms of Trp51, some of the backbone atoms of Asp133 and Pro127, W1 to W3, and a part of PhiKan5196 (P96) (Figure 3). Some of the backbone atoms were included to obtain valid QM/MM cuts (Murphy et al., 2000). The QM region was saturated with capping hydrogen atoms. The QM region of model 1 was the same as that of model 2, except for the absence of W1 to W3 (see Figure S1). In other words, there are no water molecules in the QM region of model 1. The QM region of model 2 comprised 98 atoms, while the QM region of model 1 comprised 89 atoms; the remaining MM parts were composed of 32,375 atoms in both models.

The MM region of both systems was described using the Amber ff14SB force field in MM calculations. In the QM/MM implementation, E_MM_ is calculated classically from the MM atom positions, whereas H_QM_ is evaluated using the B3LYP and LANL2DZ bases that are set for all the atom types. The AMBER16 (Case et al., 2016) integrated Gaussian 09 (Frisch et al., 2009) package was used to carry out all the QM/MM MD simulations.

The QM/MM minimization process included 500 steps of steepest descent, which was followed by 200 steps of conjugate gradient minimization. In this process, any atom within 10Å of the ligand was allowed to move freely and the remaining part of the system was kept restrained. Subsequently, the entire system was simulated for 2.5 ps by using the NPT ensemble with a time step of 0.5 fs, and the temperature was regulated using a Langevin thermostat at 300 K (Loncharich et al., 1992). The QM part of the system was not constrained by the SHAKE algorithm (Ryckaert et al., 1977). The non-bonded interactions were calculated using a cutoff of 10Å. We carried out six identical simulations for each model; therefore, a total of 3,000 snapshots (500 for each trajectory) for each model were used for subsequent analysis.

### Clustering Analysis

The snapshots of each model were classified into several clusters using the K-means clustering method (Arthur and Vassilvitskii, 2007). As a simple and robust algorithm, the K-means method is suitable for identifying significant structural features. An arbitrarily low number of clusters may lead to important information being missed, whereas too many clusters would be unnecessarily redundant. Thus, it is necessary to set an appropriate number of premeditated clusters. The number of clusters was set to three after testing the cluster numbers of two to 10 (i.e., *k* = 2~10); this was aimed at achieving a better structural similarity within each cluster. Five variables were used to serve as clustering descriptors, namely: length and angle of the halogen bond [symbolized by d(I⋯O) and ∠(C-I⋯O)], length of the three hydrogen bonds formed by three water molecules [symbolized by d(I⋯O_w1_), d(I⋯O_w2_), and d(I⋯O_w3_)]. To ensure that the descriptors were comparable to each other, we normalized the five kinds of descriptors. This protocol has been implemented with the scikit-learn (Pedregosa et al., 2011) module of Python.

### Interaction Energy Calculation

We used the Moller—Plesset second order (MP2) (Headgordon et al., 1988) method with an aug-cc-pVTZ-PP pseudo-potential basis set for iodine atoms and the aug-cc-pVTZ basis set for hydrogen, carbon, nitrogen, and oxygen atoms; this was aimed at calculating the interaction energy. The calculated interaction energies (ΔE_Corr_) of the complexes were corrected for the basis set as superposition error that employed the counterpoise method of Boys and Bernardi (1970). All *ab initio* calculations were carried out using the Gaussian 09 package (Frisch et al., 2009).

For convenience, the hydrogen-bonded complexes are denoted by A–B, and the halogen-bonded complexes are denoted by B–C; A–B–C represents the entire complexes. Here A represents the three water molecules around the iodine atom, B stands for the ligand fragment, and C denotes the leucine residue fragment. The halogen bonding interaction energies are calculated here according to the method employed in earlier studies pertaining to cooperativity effects (Zhao et al., 2011).

(1) $$\begin{array}{l} {\Delta E}_{BC}=E_{\text{ABC}}-\left({\text{E}}_{\text{C}}+\;E_{AB}\right) \end{array}$$

where E_ABC_ is the total energy of the entire system and E_A_, *E*_*C*_ and _E_BC_, *E*AB_ are the energies of the monomeric and the binary systems. It should be noted that the interaction of B and C (Δ*E*_*BC*_) also includes the interaction energy between fragments A and C. In this work, we consider Δ*E*_*BC*_ as a small constant since the distance of A and C is relatively long.

### Correlation Coefficient Matrix Analysis

To evaluate the effect of the three water molecules on the halogen bond, correlation coefficients matrices (Dawson and Trapp, 2004) were calculated between two pairs of the three hydrogen bond lengths, based on Eq. (2):

(2) $$\begin{array}{l} R_{ij}=\frac{C_{ij}}{\sqrt{C_{ii}*C_{jj}}} \end{array}$$

(3) $$\begin{array}{l} C_{ij}=\frac{1}{F}\overset{F}{\underset{i=1}{\sum }}(ri-{\bar{r}}_{i})\left(r_{j}-{\bar{r}}_{j}\right) \end{array}$$

In Eq. (2), *C* is the covariance matrix calculated between two variables, as shown in Eq. 3. *F* is the number of the total frames (or structures) and *r* is the hydrogen bond length. The values of *R* are between−1 and 1, both inclusive. Udovicic et al. (2007) stated that the absolute values of correlation coefficients from 0 to 0.25 indicate an absence of correlation, those from 0.25 to 0.50 indicate poor correlation, those from 0.50 to 0.75 indicate moderate to good correlation, and those above 0.75 indicate good to excellent correlation.

## Results

### Comparison of QM/MM MD Trajectories of Two Models

**Figure 4:**
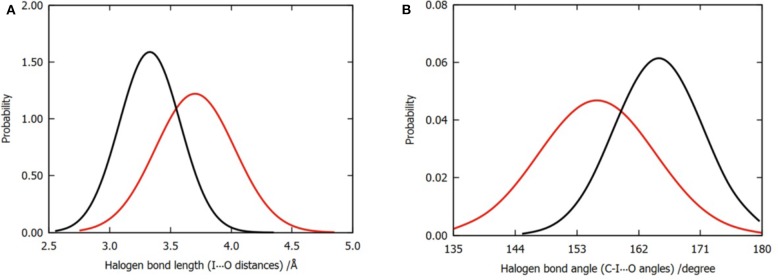
Normalized distributions of halogen bond length and angle. **(A)** Distribution of halogen bond length that is labeled by I⋯O distances. **(B)** Distribution of halogen bond angle that is labeled by C-I⋯O angles. The color codes are red for model 1 and black for model 2.

In order to compare the difference in the halogen bonds between the two models, we calculated the distribution of 3,000 frames of the I⋯O distance and the C-I⋯O angle for each model, as shown in Figures 4A,B. Evidently, the mean values of the I⋯O distance and the C-I⋯O angle (bond length and bond angle of halogen bond) are different. The average bond length was shortened from 3.70 to 3.45Å (Figure 4A); meanwhile, the bond angle was widened from 156 to 165° (Figure 4B); both of these values suggested that the three water molecules tend to enhance the halogen bond between the P96 ligand and the p53 protein (Cavallo et al., 2016). In addition to the overall distribution, the average value of the halogen bond length and the bond angle of each trajectory (Tables S1, S2) can also support the result.

### Clustering of the Trajectories and the Roles of Water Molecules

**Figure 5:**
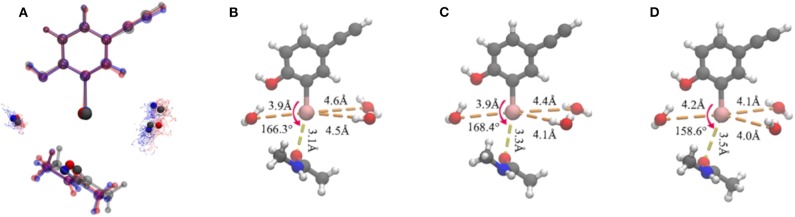
Representation of the clustering results. **(A)** Superposition of the three representative structures of each cluster in a ball-and-stick model. It is worth noting that the iodine atom, the oxygen atoms in the three water molecules, and the oxygen atom in the protein that formed a halogen bond are seen in the opaque material. The oxygen atoms in the water molecules in each cluster are shown as points. Color code: red, cluster A; gray, cluster B; blue, cluster C. The simplified representative structure of cluster A **(B)**, cluster B **(C)**, and cluster C **(D)**, saturating the broken bond with hydrogen atoms. The values of the five clustering descriptors are indicated.

In this section, we focused on analyzing the function of the three water molecules. Therefore, the majority of the analyses were based on the trajectories of model 2. The K-mean algorithm was applied with the aim of partitioning six trajectories of model 2 into distinct clusters by their geometries. The final clustering results of the 3,000 frames of these dynamic trajectories are listed in Table 1, and the representative structures of the three clusters are shown in Figure 5. We classified the frames of the trajectories into three distinct classes, after comparing its results with trials in different numbers of classes.

**Table 1:** Average values and standard deviations of key parameters and number of structures of three clusters.

**Cluster type**	**Halogen bond length (Å)**	**Hydrogen bond length** **(Å)**			**Halogen bond angle (**°**)**	**Number of structure**
	**I⋯O**	**I⋯O_**W1**_**	**I⋯O_**W2**_**	**I⋯O_**W3**_**	**C-I⋯O**	
A	3.2 ± 0.2	3.8 ± 0.2	4.5 ± 0.3	4.6 ± 0.2	168.3 ± 4.5	874
B	3.2 ± 0.2	4.0 ± 0.2	4.4 ± 0.3	4.0 ± 0.2	167.3 ± 4.9	1,094
C	3.6 ± 0.2	4.1 ± 0.2	4.0 ± 0.3	3.9 ± 0.3	159.4 ± 5.8	1,032

**Figure 6:**
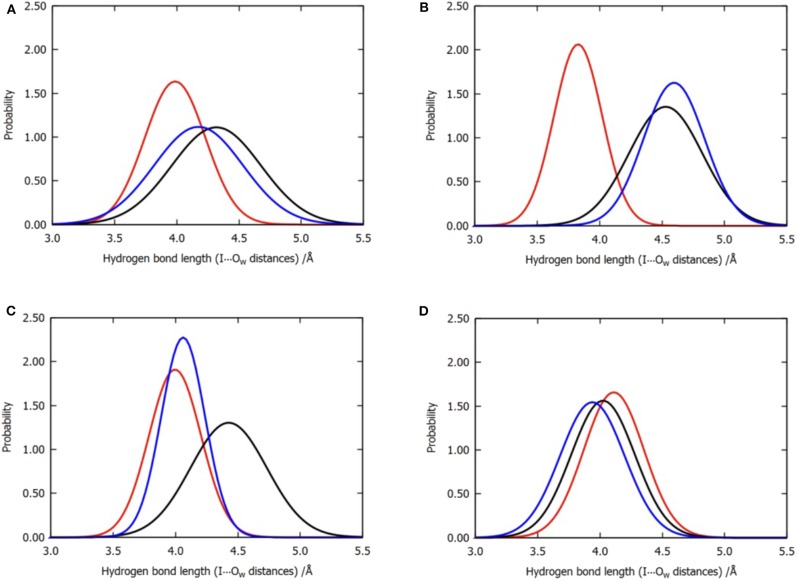
Normalized distribution of the locations of the three water molecules near the iodine atom for all the six trajectories of model 2. **(A)** Overall distributions without clustering, **(B)** cluster A, **(C)** cluster B, and **(D)** cluster C. Color code: red, W1; black, W2; blue, W3.

Several geometric characteristics of these three similarly sized clusters can be seen in Table 1. Firstly, in terms of the length and the angle of the halogen bond, the I⋯O distance and the C-I⋯O angle are similar for cluster A and B; this implies that clusters A and B have a similar halogen bond strength. The I⋯O distance is increased by 0.4Å for both average values (Table 1). The representative structure (Figures 5B,D) and the C-I⋯O angle are decreased by 7.7 and 9°, respectively, on average (Figures 5B,D) in cluster C, as compared to those in cluster A, which indicated that the average bond strength of cluster C is weaker than that of both A and B. Secondly, in terms of the length of the hydrogen bond, in cluster A W1 is significantly closer to the iodine atom than that in W2 and in W3. The main difference between clusters A and B is the I⋯O_w3_ distance (hydrogen bond length), which was reduced by 0.6Å on average (Table 1 and Figures 6B,C) and by 0.4° in the representative structure (Figures 5B,C) from clusters A to B. The main difference between clusters A and C is reflected on the distances of I⋯O_w3_ and I⋯O_w2_, which were reduced by 0.7 and 0.5Å on average (Table 1 and Figures 6B,D) and by 0.5Å in the representative structure (Figures 5B,D) from clusters A to C; therefore, it is reasonable to consider cluster A as a representation of W1 near the halogen atom, cluster B as a representation of W3 near the halogen atom, and cluster C as the representation of W2 and W3 near the iodine atom.

Interestingly, the cluster with all the three water molecules observed to be simultaneously close to the halogen atom was not observed. In principle, the polarization of multiple hydrogen bonds should facilitate the enhancement of the halogen bond, which is determined by the nature of the σ-hole. Therefore, we further analyzed the function of the three water molecules from the perspective of energy.

The halogen bond interaction energy contributions of different amounts of water molecules in each cluster are shown in Table 2. In order to calculate the interaction energy, the protein environment was omitted and only the simplified QM region (Figure 5) was considered. The contribution of each water molecule was calculated by removing other water molecules in the representative structures. When adding W1, W2, and W3 consecutively back to their corresponding locations, the contribution of each addition to the interaction energy was −1.89, −0.01, and −0.75 kcal/mol in cluster A, −0.97, −0.34, and −1.24 kcal/mol in cluster B, and −1.64, −0.13, and −0.74 kcal/mol in cluster C, respectively (Table 2). Hence, the addition of water molecules can indeed enhance the strength of the halogen bond. This observation is in accordance with those obtained in the previous studies by Carlsson et al. (2018), which stated that a hydrogen bond formed with a halogen atom can enhance the halogen bond by increasing the positive electrostatic potential of σ-hole. Moreover, it can be seen in Table 2 that the contribution of each water molecule to the halogen bond in different clusters is not consistent: the largest one in clusters A and C is from W1, whereas that in cluster B is from W3.

**Table 2:** Halogen bond interaction energy Δ*E* (kcal/mol) of the representative structure for each cluster in the presence of different water molecules and the change in the halogen bond interaction energy, Δ*E* (kcal/mol), due to the presence of additional water molecules (at the MP2/aug-cc-pVTZ-PP level of theory for I and at the MP2/aug-cc-pVTZ level of theory for H, C, N, and O).

**Cluster**	**Δ**E****			**ΔΔ**E****		
	**W_**1**_**	**W_**2**_**	**W_**3**_**	**W_**1**_-no water**	**W_**1**_W_**2**_ - W_**1**_**	**W_**1**_W_**2**_W_**3**_ - W_**1**_W_**2**_**
A	−5.61	−3.74	−4.38	−1.89	−0.01	−0.75
B	−4.43	−3.80	−4.54	−0.97	−0.34	−1.24
C	−4.71	−3.20	−3.72	−1.64	−0.13	−0.74

The observations performed with regard to energy are correlated with the previous distance analyses; however, these are also different in some aspects. In cluster C, the distance between the three water molecules and the halogen atom are similar, while their energy contributions vary from −0.13 to −1.64 kcal/mol (Table 2). Therefore, we concluded that the functions of the three water molecules in enhancing the halogen bond are not equivalent, and the order of their general contribution is given by W1 > W3 > W2; this can be reflected in the overall locations of the three water molecules in Figure 6A. This is because the position of W1 depicts that it can form a double hydrogen bond not only with the hydroxyl oxygen on the benzene ring but also with iodine atom, which in turn is stabilized by hydrogen bonds with the main-chain atoms of Val52 and Asp133 (Wilcken et al., 2012). Nevertheless, this order is not absolute. In nearly one-third of all the structures from cluster B, the contribution of W3 is observed to be greater than that of W1, which is also reflected in Figure 6C.

### Relationship of the Water Molecules

In this section, we performed various analyses to elucidate the dynamic polarization effect exerted by the three water molecules; this is achieved by analyzing the representative trajectory. The hydrogen bond lengths formed by the iodine atom and the three water molecules for each trajectory are shown in Table S1. The existence of a correlation between the movements of the three water molecules was first validated. The correlation coefficients (Eq. 3) between the pairwise hydrogen bond length of the three water molecules were calculated for 500 frames in each trajectory (Table 3).

**Table 3:** Cross-correlation coefficients between two pairs of three hydrogen bond lengths in six trajectories.

**Trajectory ID**	**w1 and w2**	**w1 and w3**	**w2 and w3**
1	−0.44	−0.49	0.37
2	−0.31	−0.58	0.12
3	−0.08	−0.29	0.70
4	−0.57	−0.28	0.33
5	−0.19	−0.22	0.16
6	−0.16	−0.13	0.26
Mean value	−0.29	−0.33	0.32

The absolute values of the pairwise correlation coefficients of the three water molecules are not >0.6, which implied that their motions are weakly correlated. In addition, the correlation between the hydrogen bonds varies with the trajectory. (Parra and Ohlssen, 2008) confirmed that two bifurcated H bonds observed to be simultaneously present in the same molecule were found to cooperatively reinforce each other from geometrical, energetic, and topological analyses, although according to the results obtained in the study by Hogan, there is no such situation wherein two water molecules led to the simultaneous formation of hydrogen bonds with halogen atoms such as W2 and W3 (Hogan and van Mourik, 2019). Structurally, in our system, W2 and W3 are on the same side of the benzene ring, and a triangular interaction network is formed between the two water molecules and the iodine atom; this facilitates the formation of this structure. Therefore, the relationship between multiple water molecules around the halogen atom is complicated and is also related to the surrounding chemical environment that needs to be studied further.

### Effect of Protein-Rich Environment on the Location of Water Molecules

**Figure 7:**
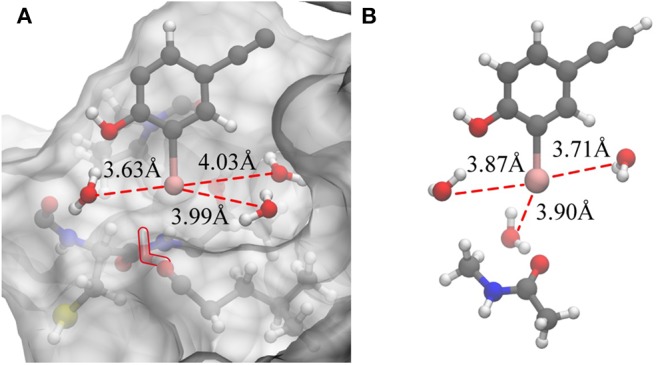
Changes in the hydrogen bonds formed by the three water molecules and the iodine atom in a protein-rich environment and under vacuum. **(A)** The QM/MM-minimized structure of model 2 in the protein environment. **(B)** The optimized simplified QM structure in vacuum (at the MP2/aug-cc-pVTZ-PP level of theory for I and at the MP2/aug-cc-pVTZ level of theory for H, C, N, and O).

Based on the previous analysis, it can be established that there is no such situation wherein three water molecules are simultaneously near the iodine atom in a protein-rich environment. For comparison, we optimized the QM structure in a protein-rich environment (QM/MM-minimized) as well as under vacuum (Figure 7). It was discovered that the three water molecules can be near the iodine atom at the same time under vacuum. In the protein environment, except for W1, the distances between the remaining two water molecules and the iodine atom are observed to be larger than those in vacuum. Therefore, the protein environment could weaken the hydrogen bonds formed by three water molecules with the incorporation of an iodine atom. Additionally, compared to the structure obtained in the protein-rich environment (Figure 7A), we found that the position of W2 in vacuum remains unchanged; however, W3 is observed to rearrange to the back of the benzene ring that is occupied by Thr135 and *vice versa* (Figure 7A). Thus, the illustrated steric constraint effects and the weakening of hydrogen bonds caused by the protein environment cause a reduction in the strength of the halogen bond in the protein-rich environment (the interaction energy of the halogen bond in vacuum is −11.91 kcal/mol and that in protein is −6.78 kcal/mol).

Consequently, we wonder whether the presence of water molecules also strengthen the halogen bonds in other halides. Therefore, we carried out a simple treatment to replace I in the optimized and simplified QM structure for both environments (protein-rich environment and under vacuum) with F, Cl, and Br. For the structure obtained after substitution, the interaction energies of halogen bonds with and without the three water molecules in both environments were calculated. It can be seen in Table 4 that other halogenated compounds exhibit the same behavior as that observed in this work, with respect to the halogen bonds; the protein-rich environment will weaken the halogen bonds, and the presence of water molecules under the same environmental condition will strengthen the halogen bonds.

**Table 4:** Halogen bond interaction energy Δ*E* (kcal/mol) of halides in the protein-rich environment and in vacuum (at the MP2/aug-cc-pVTZ-PP level of theory for I and at the MP2/aug-cc-pVTZ level of theory for H, C, N, O, F, Cl, and Br).

	**Protein environment**		**Vacuum**	
	**With three water molecules**	**Without three water molecules**	**With three water molecules**	**Without three water molecules**
F	−1.96	0.58	−7.95	0.33
Cl	−3.72	−1.04	−9.17	−1.29
Br	−4.89	−2.11	−10.08	−2.00
I	−6.78	−3.78	−11.91	−4.22

## Discussion

Herein, the role of the three water molecules around the halogen bond in the Y220C–PhiKan5196 complex of p53 protein was analyzed. In order to elucidate the polarization effect of the hydrogen bond on the halogen atom, we adopted the QM/MM MD simulation method. By comparing this method to a model that was free of water, it was revealed that the strength of the halogen bond in the model with three water molecules was stronger. This confirmed that the water molecules formed weak hydrogen bonds with halogen atoms and augmented the halogen bonds by cooperation.

The clustering and energy analyses indicated that the roles of the three water molecules were not similar, and the contribution to the cooperativity of hydrogen and halogen bonds was primarily in the following order: W1 > W3 > W2. At the same time, the protein-rich environment played an important role in the location of the water molecules.

We believe that these results provide insights into the dynamic cooperation of the hydrogen and the halogen bonds, which can facilitate the development of the force field for halogen bonds. Meanwhile, these results are also useful for rational drug design.

## Data Availability Statement

All datasets generated for this study are included in the article/Supplementary Material.

## Author Contributions

JG conceived and designed the study. TD, JG, and XW wrote the manuscript. TD, HP, and XH performed QM/MM MD simulation and data analysis.

## Conflict of Interest

The authors declare that the research was conducted in the absence of any commercial or financial relationships that could be construed as a potential conflict of interest.
