# Supplementary material for: Hybrid Molecular Dynamics for Elucidating Cooperativity Between Halogen Bond and Water Molecules During the Interaction of p53-Y220C and the PhiKan5196 Complex
Source: Front Chem. 2020 May 7;8:344. doi: 10.3389/fchem.2020.00344 (PMC7221198; doi:10.3389/fchem.2020.00344)
Supplement: Supplementary file 1 [file Data_Sheet_1.PDF]

Figure

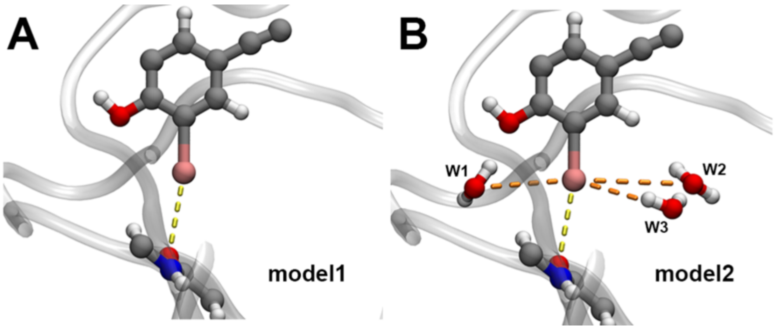

Figure S1. The simplified structures of model 1 **(A)** and model 2 **(B)**.

Tables

Table S1. Mean values of halogen bond length and bond angle, hydrogen bond length in the six trajectories of model 2, respectively

	Halogen bond length (Å)	Halogen bond angle (°)	Hydrogen bond length (Å)		
			W1	W2	W3
1	3.39	163.66	3.99	4.22	4.10
2	3.33	166.28	3.95	4.13	4.19
3	3.27	166.61	4.09	4.39	4.15
4	3.27	163.97	4.01	4.40	4.21
5	3.45	161.60	3.96	4.34	4.08
6	3.27	167.33	3.92	4.42	4.32
Mean	3.33	164.91	3.99	4.32	4.18

Table S2. Mean values of halogen bond length and bond angle in the six trajectories of model 1, respectively

	Halogen bond length (Å)	Halogen bond angle (°)
1	3.56	159.64
2	3.53	158.95
3	3.55	159.31
4	3.74	153.82
5	3.80	152.86
6	3.56	158.05
Mean	3.62	157.11
